# Variations in strength-speed-power performance across the season: do true changes occur in elite rugby players?

**DOI:** 10.5114/biolsport.2024.135201

**Published:** 2024-02-07

**Authors:** Irineu Loturco, Chris Bishop, Santiago Zabaloy, Túlio B.M.A. Moura, Maurício S. Ramos, Lucas A. Pereira, Michael R. McGuigan

**Affiliations:** 1 NAR – Nucleus of High Performance in Sport, São Paulo, Brazil; 2 Department of Human Movement Sciences, Federal University of São Paulo, São Paulo, Brazil; 3 University of South Wales, Pontypridd, Wales, United Kingdom; 4 Faculty of Science and Technology, London Sports Institute, Middlesex University, London, United Kingdom; 5 Carnegie School of Sport, Leeds Rhinos, Leeds, United Kingdom; 6 Leeds Beckett University, Headingley Campus, Leeds, United Kingdom; 7 CBRu – Brazilian Rugby Confederation, São Paulo, Brazil; 8 Sports Performance Research Institute New Zealand (SPRINZ), Auckland University of Technology, Auckland, New Zealand; 9 School of Medical and Health Sciences, Edith Cowan University, Perth, Australia

**Keywords:** Athletic performance, Team-sports, Muscle strength, Sprint speed, Resistance training

## Abstract

This study aimed to determine, through the use of a highly sensitive statistical tool, whether real changes in performance were present; and compare the rates of meaningful variations in strength, speed, and power parameters at different time-points during the competitive season in national team rugby players. Thirty-two players were assessed 5 times across the season using the following tests: squat jump and countermovement jump tests; 30-m sprint velocity; and one-repetition maximum (1RM) in the half-squat and bench-press exercises. A repeated-measures analysis of variance was conducted to test for differences between successive time-points. Individual coefficients of variation values were used to set target scores for post-measurements and examine whether changes in performance parameters were greater than the natural test variance, thus providing an indication of whether “true changes” occurred. No significant changes were detected in the vertical jump height, 1RM measures, and sprint velocity and momentum throughout the 11-month period (*P* > 0.05). True changes occurred much more frequently for strength-power measures than for sprint velocity and momentum. Elite rugby union players did not exhibit significant variations in neuromuscular performance across the competitive period, when a group-based analysis was conducted. However, at the individual level, “true changes” in strength-power-(but not in speed-) related qualities were consistently observed over the competitive season.

## INTRODUCTION

In elite sports, resistance training programs are designed to improve the physical performance of athletes, and hence, their ability to effectively execute technical and tactical actions [1, 2]. Relevant reviews have addressed this issue, concluding that there is sufficient evidence to encourage the frequent use of these strategies in athletes’ preparation [1, 2]. The importance of strength and power for athletic development may also be supported by a range of cross-sectional studies showing close associations between strength-power-related variables and various performance measures (e.g., sprint time, top-speed, and jump height), as well as their potential to discriminate between athletes from distinct competitive levels [1–5]. Whereas correlational and comparative investigations can be complementary in many respects, the lack of long-term interventions in team-sports, precludes robust conclusions about the actual effects of resistance training on athletic performance (or even in strength-power development itself) [1, 2].

Specifically in rugby union, a team-sport that relies heavily on maximum strength and power [3, 6–8], the implementation of effective strength-power training programs has a critical impact on player and team performance [3, 9, 10]. Accordingly, short-term studies (4 weeks) conducted during the competitive phase have reported increased strength (i.e., bench-press [BP] and squat 1RM) and jumping performance following different resistance training methods in rugby union players (i.e., “full-body vs. split-body resistance training” and “contrast strength-power vs. contrast speed-power training”, respectively) [9, 11]. Despite the absence of clear differences between training protocols, in both studies [9, 11], the authors highlighted and described the crucial role played by strength-power training volume (i.e., exposure to high volume-load stimuli) in modulating training-induced adaptations (specifically those related to power production).

As an additional attempt to examine the effects of resistancebased interventions and map strength-power changes across the season, studies dealing with seasonal variations are widely performed in rugby union [7, 12–14]. In a 45-week study on strength-power development in rugby union players from an English premiership team, Gannon et al. [7] observed worthwhile (i.e., estimated by magnitude-based inferences), but not statistically significant, differences in peak force and power output (i.e., assessed using isometric squat and “explosive hack-squat” exercises, respectively) throughout the competitive season. Zabaloy et al. [15] observed a similar pattern (i.e., lack of significant changes) in the evolution of measures of relative strength (squat- and BP-1RM) and squat peak force in a 10-month study in elite young rugby union players. Notably, these two studies also found small and significant decreases in rapid force production (-6.3% and -8.8% for peak force at 50 and 100 ms, respectively) [7] and sprint velocity (+4%, on average, for sprint time at 30-m) [15] in the latter stages of the season, which certainly compromise rugby-specific performance.

The lack of positive changes in sprint velocity and power-related capabilities across the season is commonplace in many sports and may be related to a number of factors [13, 15–18]. High exposure to match loads throughout the season, concurrent training effects, limited trainability for speed qualities, and low frequency and volume of sprint-specific and strength-power training are usually cited as the main barriers to adequate speed-power development [6, 17, 19–22]. The low level of transference from strength improvements to sprinting speed is another critical factor that may affect the enhancement of speed qualities throughout the season – an issue that seems even more pronounced in highly-trained rugby players (and well-trained athletes of other sports) [10, 17, 23]. Indeed, it is likely that the complexity of sprinting technique, associated with the minimal remaining trainability in athletes with an extensive training background, may further reduce the transfer potential of strength training to sprinting performance [10, 17, 23]. However, it should be emphasized that when applied to small and highly-specialized samples (i.e., elite athletes), more conventional statistical approaches may not be sensitive enough to assess meaningful changes in speed- and power-related capabilities [17, 24, 25]. In this sense, even when “real changes” occur (i.e., when variations in post-test scores are greater than pre-test variance) [17, 24, 25], they can be undetected if assessed by traditional inferential methods (e.g., null hypothesis significance testing). Thus, specifically when small (and non-significant) changes in performance are expected, the use of more sensitive statistical techniques (e.g., “true changes”) [17, 24, 25] could serve as a guide for decision-making regarding training progression, especially when aiming to adjust programming and make decisions at the individual level [24, 25].

Considering previous studies that analyzed the evolution, and confirmed the stability, of strength, speed, and power parameters in elite rugby union players across the season [7, 12–14], we believe it is important to investigate whether true changes in these measures occur at the individual level. Therefore, the objectives of this study were to: 1) determine, through the use of a more sensitive statistical tool (i.e., true changes calculation), whether real changes in strength-speed-power-related performance were present; and 2) compare the rates of meaningful changes between strength, speed, and power measures at different time-points during the competitive season in national team rugby players.

## MATERIALS AND METHODS

### Participants

Thirty-two male rugby union players from the Brazilian national team (backs: n = 13; age: 24.8 ± 2.4 years; body-mass [BM]: 91.3 ± 10.1 kg; height: 1.78 ± 0.07 m; forwards: n = 19; age: 25.7 ± 3.2 years; BM: 112.1 ± 9.3 kg; height: 1.84 ± 0.08 m) participated in this study. Players were assessed during the 2022 annual season, from January to November, and participated in a total of 19 matches, during three international tournaments (comprising 17 matches) and 2 international friendly-matches. The study was approved by the local Ethics Committee and all athletes signed an informed consent form before participating in the study.

### Study Design

This longitudinal comparative study analyzed the variations in the neuromuscular performance of national team rugby union players over an 11-month period. Athletes were assessed on 5 occasions: at the beginning of the preseason period (week 1, T1); at the end of the preparatory period and approximately 10 days before the first official match of the season (week 8, T2); after the first tournament of the year (“Super Rugby Americas”) (week 21, T3); at the end of the inter-season period (week 29, T4); and at the end of the competitive season (week 44, T5), as part of their regular physical testing routine. The testing procedures followed the schedule set by the national team’s coaching staff. All testing sessions were performed in the morning, between 09:00 and 12:00 hours, prior to the first training session of the day. During all testing sessions athletes performed on the same day and in the following order: squat jump (SJ) and countermovement jump (CMJ) tests, 30-m sprint velocity (assessed in all sessions, with the exception of T2, due to a decision of the national team coaching staff), and one-repetition maximum (1RM) in the half-squat (HS) and BP exercises. Before performing the tests, players completed a 10-min standardized warm-up, comprising 5-min of running at a moderate self-selected pace followed by 5-min of dynamic stretching for both upper- and lower-limbs. Prior to the actual measurements, athletes performed 5 submaximal trials of each specific test with a 30-s interval between each trial. The average number of weekly training sessions as well as the typical strength-power training programs followed by the national rugby players (i.e., backs and forwards) during the different phases of the annual season are presented in Tables 1, 2, and 3. Resistance training intensity was constantly monitored and adjusted throughout the season, according to the actual variations in 1RM measurements. The 1RM values collected during the first control session (5–7 days after T1) were used to calculate the CVs for the 1RM measurements. During the study, nutrition and sleep habits of the athletes were monitored by the technical staff of the Brazilian national team.

**TABLE 1:** Average number of training sessions for backs and forwards and total number of matches played during the different phases of the annual training season.

		Training Phase			

		T1–T2 Preseason	T2–T3 CP1	T3–T4 Inter-Season	T4–T5 CP2
**Strength-power training**	*Backs*	3.2 ± 1.1	1.8 ± 1.9	4.0 ± 1.1	3.4 ± 0.6
	*Forwards*	2.8 ± 1.3	2.2 ± 0.4	3.0 ± 1.4	3.7 ± 1.3

**Anaerobic and aerobic powe**r	*Backs*	2.4 ± 0.5	1.0 ± 0	0.5 ± 0.5	0.4 ± 0.5
	*Forwards*	1.6 ± 0.9	1.0 ± 0	0.5 ± 0.5	0.4 ± 0.5

**Game-based training**	*Backs*	0.8 ± 0.4	0.9 ± 0.7	1.5 ± 0.5	1.7 ± 0.5
	*Forwards*	1.0 ± 0	1.2 ± 0.4	1.5 ± 0.5	1.7 ± 0.5

**Speed training**	*Backs*	0.4 ± 0.4	0.9 ± 0.9	1.0 ± 0	1.0 ± 0
	*Forwards*	0.6 ± 0.5	1.0 ± 0	1.0 ± 0	1.0 ± 0

**Technical-tactical trainin**g	–	5 (90–120’)	4 (60–90’)	4 (90–120’)	4 (60–90’)

**Official matches**	–	0	10	0	9

*Note:* CP1 (Competitive phase 1): comprised the “Super Rugby Americas Tournament”; CP2 (Competitive phase 2): comprised the “South America Rugby Championship” and the international matches; Strength-power training sessions involved traditional (e.g., halfsquat, bench-press, prone row, etc.), ballistic (e.g., jump squat, medicine ball throw, etc.), and plyometric (e.g., drop and hurdle jumps, and bounding) exercises; Anaerobic and aerobic power training sessions involved small-sided games and high-intensity interval training; Game-based training involved sessions designed to improve physical and technical skills according to playing positions; Speed training sessions involved traditional and resisted sprints (sled pushing and sled pulling), and technical drills (e.g., skipping and highknee running); Technical-tactical training sessions involved simulated rugby matches and specific tactical tasks. For technical-tactical training, the numbers in parenthesis correspond to total volume of training sessions in minutes.

**TABLE 2:** Typical strength-power training programs prescribed for backs and forwards during the preseason period.

	Backs				Forwards			

	Exercise^*^	Sets	Reps	Load^#^	Exercise^*^	Sets	Reps	Load^#^
**Day 1**	Jump squat	4	6	60–70%	Isometric mid–thigh pull	3–4	10”	–
	Drop jump	4	4	45 cm	Hang high pull	4	8	70–80%
	Hurdle jump	4	3	75 cm	Drop jump	3	4	45 cm
	Snatch	4	6	60–70%	Jump squat	4	4–6	80%
	Power clean	4	6	60–70%	Push press	4	8	70–80%
	Push press	4	6	60–70%				

**Day 2**	Overhead squat	2	8–10	70–80%	Isometric squat	4	10”	–
	Unilateral row	2	8–10	70–80%	Half squat	5–6	3–4	85–95%
	Half squat	3	4–6	80–90%	Pull up	5	3–4	85–95%
	Pull up	3	4–6	80–90%	Prone row	4	3–4	85–95%
	Prone row	3	6–8	75–85%	Lunges	4	4–6	80–90%
	Lunges	3	6–8	75–85%				

**Day 3**	Reverse fly	2	10	70–80%	Push press	5	6–8	80–90%
	Biceps curl	2	10	70–80%	High pull	5	6–8	80–90%
	Push press	3	6	60–70%	Parallel dips	4	8	+25% BM
	High pull	3–4	6	60–70%	Reverse fly	4	10	70–80%
	Lateral raises	3	6–8	75–85%	Shoulder press	3–4	8–10	70–80%

**Day 4**	Bench press	2–3	8–10	75–80%	Isometric bench press	4	5”	–
	Stiff	3	4–6	80–90%	Nordic	4	4	BM
	Push up	3	4–6	80–90%	Bench press	5	3–4	85–95%
	Bench press 45^o^	3	4–6	80–90%	Deadlift	5	3–4	85–95%
	Nordic	3	6–8	BM	Prone row	4	6–8	80–90%
					Hip thrust	5	6–8	80–90%

*Note:*

* In general, 2–3 minutes were provided between exercises and sets.

# Percentage of one-repetition maximum (i.e., mean values reported by the technical staff); BM: body-mass.

**TABLE 3:** Typical strength-power training programs prescribed for backs and forwards during the competitive period.

	Backs				Forwards			

	Exercise^*^	Sets	Reps	Load^#^	Exercise^*^	Sets	Reps	Load^#^
**Day 1**	Jump squat	3–4	6	45%	Jump squat	4	6–8	70–80%
	Drop jump	3	4	45 cm	Box jump	3	4–6	45–60 cm
	Hurdle jump	3	4	75 cm	Isometric mid–thigh pull	3–4	10”	–
	Clean and jerk	4	6	60%	Drop jump	3	4–6	45 cm
	Med ball throw	4	4	10 kg	Push press	4	4–6	80–90%
	Push press	4	6	60%	Med ball throw	3	4–6	12–15 kg

**Day 2**	Bench press	3	4–6	80–90%	Bench press	4	2–3	>90%
	Unilateral stiff	3	4–6	80%	Deadlift	4	2–3	> 90%
	Incline fly	3	4–6	80%	Bench press 45o	3	4–6	85–90%
	Med ball throw	4	4–6	10 kg	Med ball throw	3	4–6	12–15 kg
	Nordic	3	4–6	BM	Hip thrust	4	4–6	80–90%
	Hip thrust	3	6	60–70%	Leg curl	3–4	6–8	70–80%

**Day 3**	Pull-up	3	6	70%	Pull–up	3	4–6	> 80%
	Jump squat	3	4–6	60%	Half squat	4	2–3	> 90%
	Drop jump	3	6	> 45 cm	Jump squat	4	4–6	80–90%
	Bench press 45o	3	4–6	80–90%	Prone row	4	6–8	80–90%
	Bulgarian squat	3	6	60–70%	Lunges	3	4–6	> 85%
	Bounding	3	6	BM	Drop jump	3	5	45 cm

*Note:*

* In general, 2–3 minutes were provided between exercises and sets.

# Percentage of one-repetition maximum (i.e., mean values reported by the technical staff); BM: body-mass.

### Procedures

#### Vertical Jump Tests

Vertical jump height was assessed using the SJ and CMJ. In the SJ, a static position with a ~90° knee flexion angle was maintained for 2-s before a jump attempt without any preparatory movement. In the CMJ, players were instructed to perform a downward movement followed by complete extension of the lower limbs and the amplitude of the countermovement was freely determined to avoid changes in the jumping coordination pattern. All jumps were executed with hands on the hips. Five attempts at each jump were performed interspersed by 15-s intervals. The jumps were performed on a contact platform (Elite Jump System^®^; S2 Sports, São Paulo, Brazil) and the best attempt at each jump was used for data analysis purposes.

### Sprint Velocity

Sprint testing was conducted on an indoor running track. Three pairs of photocells (Elite Speed System^®^; S2 Sports, São Paulo, Brazil) were positioned at the starting line and at the distances of 10- and 30-m. Players sprinted twice, starting from a standing position 0.5-m behind the starting line. Sprint velocity was calculated as the distance travelled over a measured time interval. A 5-min rest interval was allowed between trials and the fastest time was considered for analysis. Sprint momentum (kg·m·s^−1^) was obtained by multiplying the athlete’s BM by the respective velocity (i.e., 10- or 30-m) in the sprint test.

### One-Repetition Maximum Tests in the Bench Press and Half-Squat Exercises

The 1RM test was performed using an Olympic barbell for the BP and HS exercises, as described previously [26, 27]. The testing protocol was adapted from the procedures proposed by Brown and Weir [28]. Prior to testing, athletes performed 3 specific warm-up sets. In the first set, subjects performed 4 repetitions with 50% of the estimated 1RM (i.e., based on prior assessments); in the second set they performed 3 repetitions with 60% of the estimated 1RM, and in the third set they performed 2 repetitions with 70% of the estimated 1RM. A 3-minute rest interval was allowed between sets. Three minutes after the warm-up, participants were allowed up to 5 attempts (~ 80%, 90%, 95%, and [1–2 repetitions] > 95% of the estimated 1RM) to obtain the 1RM load (e.g., maximum weight that could be lifted once using the proper technique) [28], with a 3-minute interval between attempts. To account for differences in the BM of the rugby players, values were normalized by dividing the 1RM load value by the athlete’s BM (i.e., relative strength, RS).

### Statistical Analysis

Data are presented as mean ± standard deviation. Data were analyzed using the intention-to-treat approach, considering all observed data collected from the participants, according to their respective groups. The Shapiro-Wilk test was used to confirm the normality of the data. To analyze the differences in the physical tests executed across the time-points between backs and forwards, a two-way repeated-measures analysis of variance was conducted followed by the Bonferroni post hoc. Statistical significance was set as *P* < 0.05. Effect sizes were calculated using Cohens’ *d* [29] with associated 95% confidence intervals (CI). All tests used demonstrated small errors of measurement, as evidenced by their high levels of accuracy and reproducibility (i.e., coefficient of variation < 10% and intraclass correlation coefficient [using an alpha 2-way mixed model] > 0.90; for all tests). Statistical power was calculated by using the G*Power software (v. 3.1.9.7), for the different comparisons performed in the tested variables. CV values were calculated for each athlete at the individual level, and utilized to establish “target scores” for comparisons with post-testing results (i.e., T2, T3, T4, and T5) [25]. This analysis aimed to determine whether fluctuations in strength, speed, and jump parameters were greater than their “natural variability” estimated at baseline (i.e., T1), thereby providing an indication of whether true changes occurred on an individual basis [17, 24, 25].

## RESULTS

**FIG. 1:**
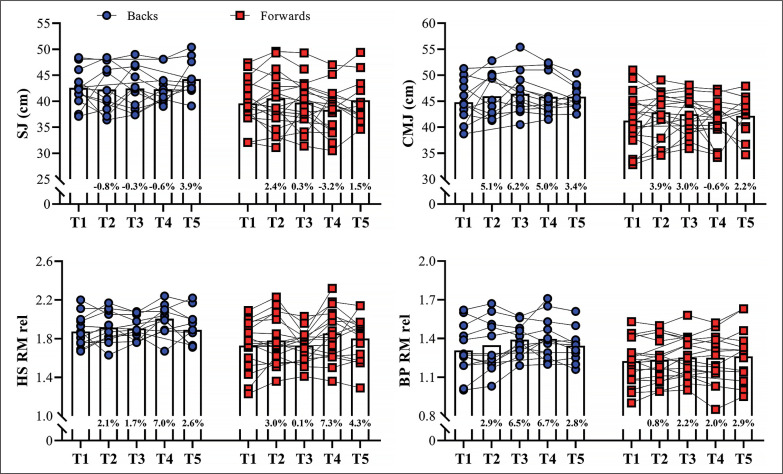
Variations in squat and countermovement jump height (SJ and CMJ) and relative values of maximum strength in the half-squat (HS) and bench-press (BP) exercises across the different time-points. RM: repetition maximum. Bars represent mean values; symbols represent individual results. Percentage differences are shown in each column relative to the first assessment.

The statistical power achieved for the different comparisons, given the sample size of 32 rugby players, and considering an alpha level of 5%, was > 80%. Backs exhibited higher relative strength qualities, better sprint and jump performance, and lower sprint momentum compared to the forwards, across the 5 testing sessions (ES [95%CI] ranging from 0.66 [0.04; 1.25] to 1.69 [0.83; 2.46]; *P*-values ranging from < 0.001 to 0.043). Similar non-significant variations in the BM of backs and forwards were observed over the 11-month period (ES [95%CI] ranging from 0.05 [-0.73; 0.84] to 0.30 [-0.55; 1.13]; *P*-values ranging from 0.234 to 1.00, for backs; and ES [95%CI] ranging from 0.06 [-0.68; 0.79] to 0.38 [-0.33; 1.07]; *P*-values ranging from 0.105 to 1.00, for forwards, for main effect of time; *P* = 0.347 for group*time interaction). Figure 1 depicts the variations in vertical jump performance and relative strength in the HS and BP exercises across the 5 time-points. No significant changes were noticed for backs and forwards throughout the 11-month period in vertical jump measures and RS in both exercises tested (ES [95%CI] ranging from 0.01 [-0.84; 0.84] to 0.45 [-0.39; 1.27]; *P*-values ranging from 0.563 to 1.00, for backs; and ES [95%CI] ranging from 0.01 [-0.68; 0.68] to 0.45 [-0.25; 1.15]; *P*-values ranging from 0.168 to 1.00, for forwards, for main effect of time; *P* = 0.921, 0.657, 0.705, and 0.250 for SJ, CMJ, HS RS, and BP RS, respectively, for group*time interaction).

**FIG. 2:**
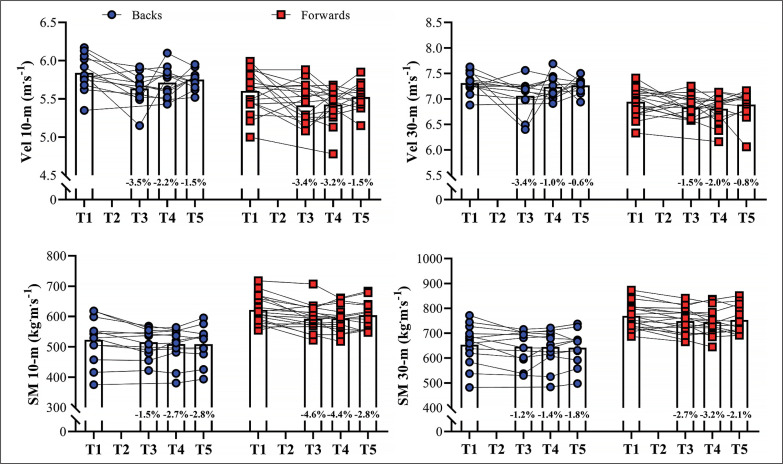
Variations in sprint velocity (Vel) and momentum (SM) at 10- and 30-m across the different time-points. RM: repetition maximum. Bars represent mean values; symbols represent individual results. Percentage differences are shown in each column relative to the first assessment.

**FIG. 3:**
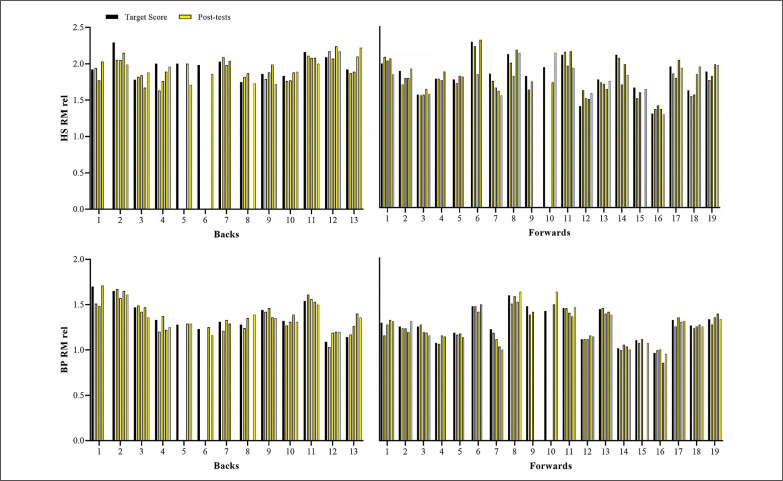
Individual comparisons between target scores and subsequent performance scores for relative values of maximum strength in the half-squat (HS RM rel) and bench-press (BP RM rel) exercises.

**FIG. 4:**
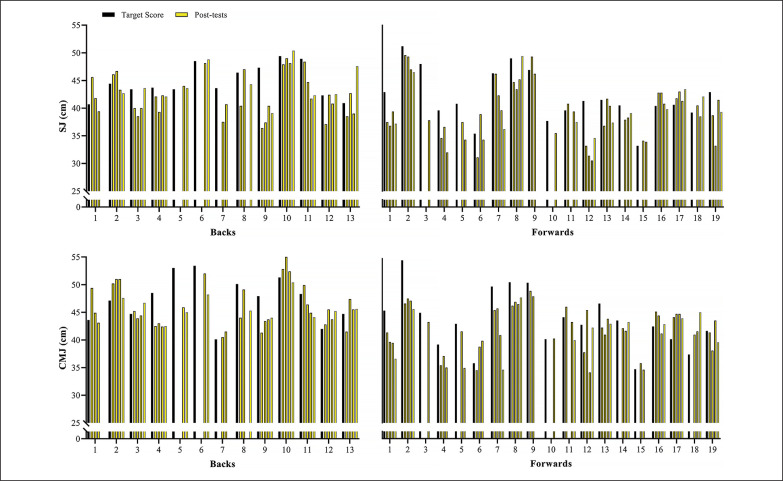
Individual comparisons between target scores and subsequent performance scores for squat and countermovement jump height (SJ and CMJ).

**FIG. 5:**
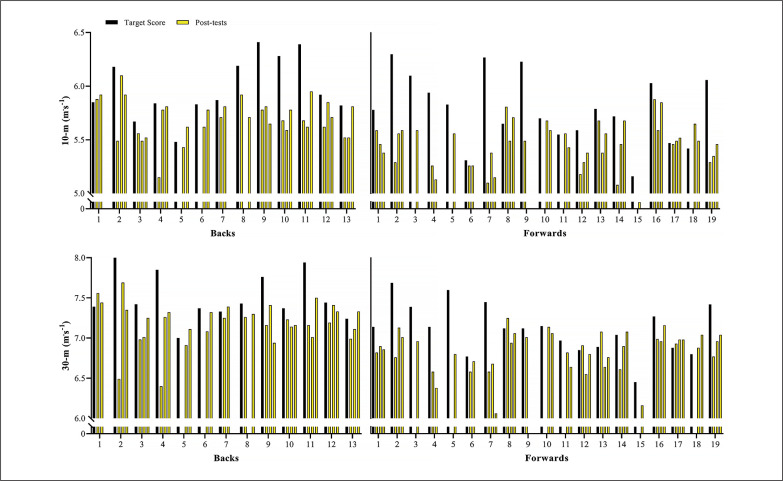
Individual comparisons between target scores and subsequent performance scores for sprint velocity at 10- and 30-m.

**FIG. 6:**
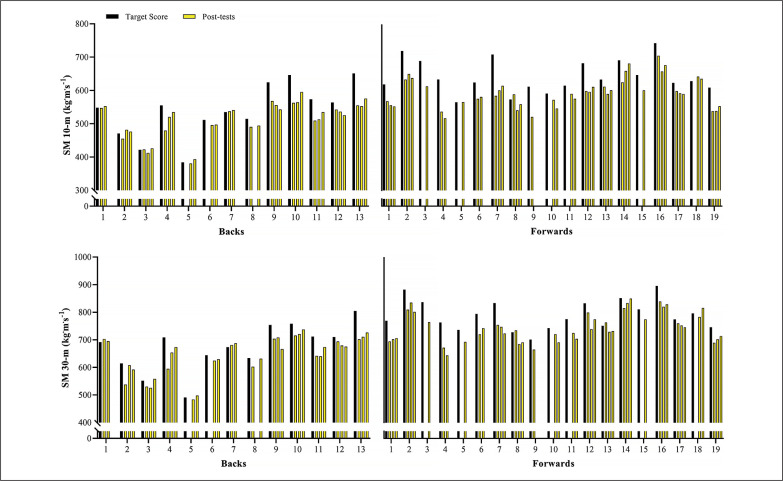
Individual comparisons between target scores and subsequent performance scores for sprint momentum (SM) at 10- and 30-m.

Figure 2 shows the variations in the sprint velocity and momentum over 4 testing occasions. No significant variations were observed for backs and forwards in these velocity-based measurements comparing the distinct assessments performed (ES [95%CI] ranging from 0.01 [-0.82; 0.81] to 0.55 [-0.29; 1.35]; *P*-values ranging from 0.113 to 1.00, for backs; and ES [95%CI] ranging from 0.02 [-0.74; 0.71] to 0.64 [-0.10; 1.34]; *P*-values ranging from 0.075 to 1.00, for forwards, for main effect of time; *P* = 0.587, 0.128, 0.499, and 0.416 for 10-m and 30-m sprint velocity and momentum, respectively, for group*time interaction). Figures 3–6 show the individual comparisons between the target scores, and the subsequent performance tests for the multiple variables analyzed. When a given athlete presented a test result higher than the target score in a specific variable, a “true change” occurred.

## DISCUSSION

We analyzed the changes in physical performance of elite rugby union players at different time-points, during an annual training season. Overall, these athletes did not exhibit significant variations in their strength-, speed-, and power-related qualities, which reinforces previous findings indicating the long-term stability of these measures across the competitive period [7, 12–14, 17, 30]. On the other hand, this highlights the need to use more sensitive statistical techniques for the assessment and evaluation of highly-trained athletes, especially when the intention is to create more effective and tailored training programs.

The lack of significant changes in neuromuscular performance throughout the competitive phase is not novel, either in elite rugby union or other team-sports [6, 14, 30]. For example, studies in soccer [16, 31, 32] and cricket [30] showed that the high demands of the competitive period associated with a multitude of in-season training practices (e.g., technical-tactical preparation, match-recovery, and injury prevention strategies, etc.) do not provide an appropriate and effective stimulus to improve strength and power qualities. Indeed, even when adequate and feasible volumes of resistance training are completed within the training schedule (e.g., 2–3 sessions of 30–45 min per week), in-season variations in independent measures of strength and power tend to be marginal in elite rugby union players [6, 14]. Nevertheless, according to our results, these relatively small (and non-significant) differences can represent meaningful changes in performance, and therefore should be considered by coaches when preparing training programs and planning individual workouts in the context of high-performance sports. It is crucial to mention that these strategies could be further enhanced in terms of effectiveness and precision by incorporating the analysis of additional jump metrics (e.g., peak and time to peak power, peak force, propulsive impulse, etc.) when utilizing force platforms for data collection [25, 33, 34].

Notably, different from the vast number and relative frequency of true changes found in strength-(BP 1RM and HS 1RM) and power-related (SJ and CMJ) qualities, sprint velocity and momentum practically did not vary across the season. Specifically, whereas more than 75% and 60% of backs and forwards exhibited meaningful variations in 1RM strength and vertical jump height, only 15% and 25% of them presented real changes in 10- and 30-m sprint velocity (Figures 3, 4, and 5; respectively). As a consequence of the lack of meaningful increases in sprint velocity (and BM), only a small percentage of players (< 15%) displayed increased levels of sprint momentum, in both 10- and 30-m (Figure 6). This is also surprising, given that general and sport-specific training programs differ considerably between playing positions (Table 1). For example, backs typically execute more explosive and strength-power oriented training sessions, with forwards exposed to more maximum strength and hypertrophy-oriented loads across the season (Tables 2 and 3). Indeed, when it comes to the development of sprinting skills in highly-trained athletes during the competitive period, coaches should keep in mind two basic concepts: 1) the narrow “window of development” for speed-related abilities, and 2) the limited transference of strength-power training to sprint performance [10, 17, 30]. It is worth noting that this is an issue that affects not only team-sport players, but also highly-specialized and powerful track and field athletes (e.g., sprinters and jumpers) from different levels, who follow tailored training programs and compete under different circumstances [17, 35–37].

As part of a long-term study, elite rugby union players were tracked over a 1-year period to examine the potential transference of lower-body strength and power gains to sprinting kinematics [10]. After comparing pre- and post-test scores, the authors concluded that the evolution of strength, speed, and power qualities over the season tend to be minimal (or absent) in rugby union players with extensive training backgrounds [10]. Moreover, even when moderate improvements in certain strength measures occur and considerable levels of transference are achieved (e.g., increases in power clean strength corresponding to increases in stride length during the acceleration phase of sprint running), these athletes do not exhibit significant changes in sprint velocity [10]. On the basis of these findings, the authors suggested that highly-trained rugby players may reach a point of “diminishing returns”, where high levels of strength do not necessarily result in positive transfer to sprinting ability [10]. Another possible explanation for the low degree of transference of strength to and lack of meaningful variations in sprint performance may be related to the high levels of physical and physiological strain that these athletes are exposed to throughout the competitive season [38, 39]. It is likely that the “excessive” training and match load accumulated during the season may negatively affect the magnitude of neuromuscular adaptations in senior rugby athletes, especially those associated with the development of complex sprint-specific skills. These excessive workloads, combined with inadequate recovery time and a gradual decrease in the relative volume and frequency of neuromuscular training, may not only hamper the proper development of speed-related capabilities within the season, but also preclude their progressive evolution across different age-categories (e.g., no differences in 10- and 40-m sprint times between junior and senior rugby players) [39, 40]. Importantly, this paradoxical finding (i.e., lack of evolution in sprint performance throughout the players’ specialization process) has also been reported for other team-sports [41, 42], and certainly deserves further investigation. Together, these factors could help to explain why the vast majority of our athletes did not exhibit any meaningful variations (i.e., true changes) in sprint velocity and momentum over the course of the annual training cycle, even when a very sensitive statistical technique was used to test for differences across multiple time points.

In summary, we demonstrated that a large proportion of elite rugby union players involved in a supervised 11-month in-season training program presented real changes in maximum strength and power-related performance. In contrast, a small number of these players improved sprint velocity and sprint momentum at 10- and 30-m. As in other studies with a similar design, this research is limited by the impossibility of manipulating and controlling training variables and match schedules, as well as the difficulty of testing the effects of specific training components (e.g., resistance or sprint-specific training sessions). However, the present study highlights the need to consider new approaches for assessing variations in the neuromuscular performance of elite athletes throughout the annual training season. Specifically, while meaningful changes (i.e., when the improvement is greater than the individual target score [25]) in strength-power qualities are frequently observed, the same does not hold true for speed-related qualities during the annual training season. These findings have important implications not only for a more accurate assessment of seasonal variations in performance, but also for the development of more effective strength, speed, and power training strategies for elite athletes. Future studies using the same statistical approach (i.e., true changes) and encompassing a larger number of jump metrics (e.g., comparing concentric and eccentric jumping parameters, asymmetries, etc.) are also needed to better understand how these parameters may fluctuate throughout the season.

## CONCLUSIONS

During the annual training cycle, national team rugby union players usually exhibit meaningful increases in 1RM strength and power-related capabilities, but rarely present real changes in sprint velocity and sprint momentum. To detect these small but important variations, practitioners are advised to utilize the “true changes” calculation by setting “target scores” and examining whether fluctuations in performance metrics are greater than their “natural variability” (estimated by the individual CVs). This statistical procedure is extremely useful when non-significant differences in physical performance are highly expected, as is the case for changes associated with strength, speed, and power performance in elite rugby union players (or other team-sport athletes) over the competitive period [7, 12, 17, 30]. Under this realistic scenario, the use of this simple and applied statistical tool can help coaches and sport scientists to make well-informed decisions on individualized training prescription and training load management. This may be particularly relevant for the creation of more effective, customized, and realistic sprint-specific training programs. Practitioners from other team-sports (e.g., soccer, futsal, rugby sevens, and cricket) could also benefit from the application of the same statistical approach given the similar pattern of strengthpower and, specifically, speed development throughout the season.

## Conflict of interest

The authors declare no conflict of interest.
